# RAS Mutation Status Should Not Be Used to Predict Outcome from Cytoreductive Surgery and Hyperthermic Intraperitoneal Chemotherapy for Colorectal Peritoneal Metastases

**DOI:** 10.1245/s10434-022-12704-9

**Published:** 2022-11-18

**Authors:** Dilraj Bhullar, Sarah O’Dwyer, Malcolm Wilson, Mark P. Saunders, Rohit Kochhar, Jorge Barriuso, Omer Aziz

**Affiliations:** 1 Division of Cancer Sciences, School of Medical Sciences, Faculty of Biology, Medicine and Health, The University of Manchester, Manchester, UK; 2 Colorectal and Peritoneal Oncology Centre, The Christie NHS Foundation Trust, Manchester, UK

## Abstract

**Background:**

Genetic biomarkers guide systemic anti-cancer treatment (SACT) in metastatic colorectal cancer. It has been suggested they have a role in selecting patients with colorectal peritoneal metastases (CRPM) for cytoreductive surgery (CRS) with hyperthermic intraperitoneal chemotherapy (HIPEC). This study aims to quantify the effect of mutation status on overall survival (OS), adjusting for confounders such as pre-operative systemic anticancer treatment (SACT).

**Patients and Methods:**

Retrospective analysis of patients undergoing CRS/HIPEC for CRPM at a national peritoneal tumour centre (2004–2017) was performed. Demographics, treatment history and operative data were extracted. Known biomarker gene mutation status was noted including: *KRAS*, *NRAS*, *BRAF*, *PIK3CA* and *MMR*. Cox regression analysis and Kaplan–Meier curves were used to determine overall survival.

**Results:**

One hundred ninety-five patients were included. Median follow-up time was 34.7 months (range 5.4–184.9 months) and median OS was 38.7 months (95% CI 32.4–44.9 months). Biomarker status was as follows: *KRAS* (*n* = 114), *NRAS* (*n* = 85), *BRAF* (*n* = 44), *PIK3CA* (*n* = 15) and *MMR* (*n* = 21). Mutation rates were 45.6%, 3.5%, 13.6%, 13.3% and 14.3%, respectively. Seventy-four per cent underwent complete cytoreduction (CC = 0), 81% received SACT pre-CRS/HIPEC and 65% post-CRS/HIPEC. RAS (*p* = 0.21) or *BRAF* (*p* = 0.109) mutation status did not predict OS. Nodal involvement, extramural vascular invasion, Peritoneal Cancer Index (PCI) score, CC score, SACT post-HIPEC and *NRAS* mutation were significant negative predictors of OS in univariate analysis (*p* < 0.05). Multivariate Cox regression confirmed CC-score > 1 (HR: 7.599, 95% CI 3.402–16.974, *p* < 0.0001) as a negative predictor of OS. RAS mutation status did not affect outcome (HR: 1.682, 95% CI 0.995–2.843, *p* = 0.052).

**Conclusions:**

RAS mutation status should not in isolation be used to select patients for CRS/HIPEC.

The RAS family of oncogenes have been shown to contribute to the development and progression of colorectal cancer (CRC),^1^ with mutations resulting in dysregulated cellular proliferation downstream of epidermal growth factor receptor (EGFR). As a result, RAS mutated CRC is resistant to anti-EGFR therapies. *KRAS* is the predominant mutated isoform in CRC (86%), whereas *NRAS* mutations are infrequent (14%) and *HRAS* mutations have not been detected.^2^ Genomic RAS mutations (in both *KRAS* and *NRAS*) are thought to be present in up to 47% of colorectal cancers.^3,4^

Colorectal peritoneal metastases (CRPM) are associated with a significantly lower overall survival (OS) compared with other CRC metastatic sites such as the liver or lung.^5^ The median OS for patients with isolated CRPM (with peritoneum as the only site of disease) is 16.3 months compared with 19.1 months with isolated liver and 24.6 months with isolated lung metastases.^6^ Cytoreductive surgery (CRS) with curative intent in a highly selected patient population followed by administration of hyperthermic intraperitoneal chemotherapy (HIPEC) is a treatment that has been shown to significantly improve OS to 46 months compared with 13.2 months with conventional surgery and systemic chemotherapy.^7^ A recent randomised controlled trial of 265 CRPM patients has demonstrated OS of 41.3 months with CRS alone compared with 41.7 months with CRS/HIPEC (*p* = 0.99) using an oxaliplatin-based regime, suggesting most of this benefit comes from the CRS component.^8^

As genomic biomarker testing has become more available, several studies of varying sizes have reported on the prognostic impact of RAS mutational status in patients undergoing CRS/HIPEC for CRPM. None of these studies have, however, considered the impact of pre-operative systemic anti-cancer treatment (SACT) on their findings, which is important as it potentially introduces a selection bias. Schneider et al. (2018) retrospectively reviewed data on CRPM patients undergoing CRS/HIPEC from six European centres, finding 186 RAS/RAF mutation patients had reduced OS compared with 192 RAS/RAF wild type patients.^9^ Morgan et al. (2019) compared 22 RAS mutant versus 23 RAS wild-type CRS/HIPEC patients, finding RAS mutation was associated with decreased recurrence-free survival of 5.4 versus 12.5 months and no significant difference in OS.^10^ Arjona-Sanchez et al. (2019) compared 36 patients undergoing CRS/HIPEC for CRPM with RAS mutations to 39 RAS wild-type patients, finding RAS mutation independently predicted OS (HR: 2.024; *p* = 0.045).^11^ Two recent studies have, however, raised a question on the role of RAS mutational status on predicting outcome from CRS/HIPEC. Graf et al. (2020) reported on 111 patients (51 *KRAS* mutant versus 60 *KRAS* wild type), finding no difference in OS between *KRAS* mutant and wild-type tumours.^12^ Baratti et al. (2021) recently reported on 152 CRPM patients selected to undergo perioperative systemic chemotherapy and CRS with or without HIPEC.^13^ No difference was reported in OS in wild-type *KRAS/NRAS/BRAF* patients compared with patients with *KRAS* or *NRAS* mutations and wild-type *BRAF* (49.7 versus 49.3 months). Both studies identified BRAF mutation alone as a marker of poor prognosis in their patient groups.

SACT an any point prior to CRS/HIPEC (which most of these patients receive), introduces a selection filter which may change the prognostic ability of the RAS mutation status to determine outcome from the procedure. This study aims to determine the impact of genomic biomarker status (*KRAS*, *NRAS*, *BRAF*, *PIK3CA* and *MMR)* on OS in patients undergoing CRS HIPEC for CRPM at a UK national peritoneal tumour centre, considering SACT, and adjusting for potential confounders using multivariate analysis.^7^

## Patients and Methods

### Patient Population

A retrospective review was conducted of all patients treated with CRS/HIPEC for CRPM at a UK national peritoneal tumour centre between 2004 and 2017. Patients were identified from a prospectively collected database and included in this study if they had CRS/HIPEC for CRPM, with confirmed primary CRC and CRPM samples. Patients with no histologically confirmed peritoneal metastasis at surgery were excluded, as were cases where the peritoneal metastasis was found to originate from a non-colorectal malignancy. All patients were discussed at a specialised peritoneal tumour multi-disciplinary team (MDT) meeting with CRS/HIPEC offered as a treatment with curative intent. Non-resectable extra-peritoneal metastatic disease and nodal disease outside the primary field was a contraindication to treatment.

### Operative Technique

The approach to CRS at our institution during the study period was with HIPEC being administered using a semi-closed colosseum technique that has been described previously.^14^ The choice of HIPEC agent was decided by specialist medical oncologists who were core members of the peritoneal tumour MDT. In all cases, this decision involved a review of the patients’ health record and treatment history, noting toxicities to previous systemic anti-cancer treatments. Following MDT review, patients received either mitomycin C- or oxaliplatin-based therapy delivered at a temperature between 42 and 43°C. Mitomycin C (35 mg/m^2^) was perfused into the peritoneal cavity for a total duration of 90 min (in three doses every 30 min) and oxaliplatin (368 mg/m^2^) for 30 min. Treatment with oxaliplatin included intravenous infusion of 5-fluorouracil (5FU) and a bolus of folinic acid 1 h before the introduction of HIPEC.

### Data Collection

All patients had their pathology reports, operation notes and hospital records reviewed. Patient demographics and treatment history (prior surgery or chemotherapy) were extracted. Operative data included date of CRS/HIPEC procedure, peritoneal cancer index (PCI) and complete cytoreduction (CC) scores at CRS/HIPEC. Intra-operative PCI scores were divided into <11, 11–15 and >15, in line with the Prodige 7 randomised controlled trial.^8^ Pre-operative tumour markers included carcinoembryonic antigen (CEA), cancer antigen (CA)125 and carbohydrate antigen (CA)19-9. Pathological assessment included features of the primary tumour, mucinous components and/or signet ring cell morphology, degree of differentiation and presence of nodal metastases (N-stage).

### Biomarker Analysis

Mutation analysis was undertaken on either the primary or metastatic tumour at accredited medical laboratories, and was arranged either by our institution or the referring hospital protocols with the intention to optimise chemotherapy options either before or after CRS/HIPEC. A pyrosequencing-based assay was primarily used to identify alterations within *KRAS* (codons 12, 13, 61, 117 and 146), *NRAS* (codons 12, 13 and 61), *BRAF* (V600) and *PIK3CA* (codons 542 and 1047) genes. To be considered ‘RAS mutant’ or ‘RAS wild type’ both *KRAS* and *NRAS* status had to reflect this status. Under standard conditions, assays were capable of detecting 5–10% mutant admixtures in a background of wild type in formalin fixed and paraffin embedded (FFPE) tissue specimens. Deficiencies in *MMR* were identified using immunohistochemistry (IHC) and staining for the expression of MLH1, MSH2, MSH6 and PMS2 proteins. All samples were required to have a tumour cell content of > 20% for mutation analysis.

### Follow-Up and Outcome Measures

Patients were followed-up every 6 months for 2 years after CRS/HIPEC and annually thereafter, with computed tomography (CT) thorax/abdomen/pelvis at 6, 12, 18, 24, 36, 48 and 60 months, accompanied by tumour markers. The primary outcome measure was overall survival (OS) from the time of CRS/HIPEC. All-cause mortality data were obtained from the UK National Cancer Registry and linked to our institutional data set.

### Statistical Analysis

Clinical, histopathological and survival data for the CRS/HIPEC cohort were collated and subjected to appropriate statistical analysis under the guidance of an experienced statistician. Univariate and multivariate Cox proportional hazards regression analysis and Kaplan–Meier curves were used to assess the impact of patient- and tumour-related characteristics on survival outcomes. Log rank testing was used to supplement results obtained from Kaplan–Meier curves and investigate survival distributions. Chi-square tests were also used to directly compare different subgroups of the population. A *p*-value of < 0.05 was chosen to represent significance. All statistical analyses and calculations were performed using SPSS version 25 software (IBM Corporation, Armonk, NY, USA).

## Results

**Fig. 1.**
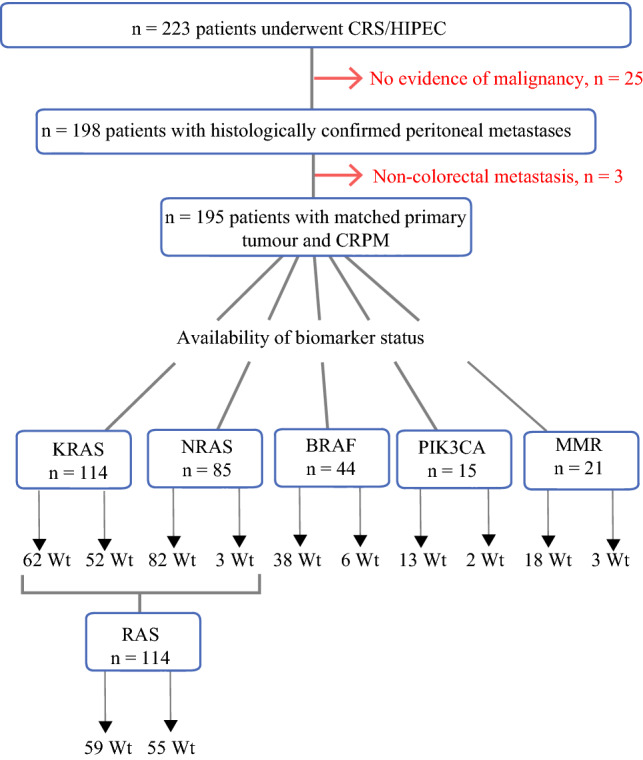
Flow diagram of patient selection and the availability of biomarker data within the study population

Between January 2004 and December 2017, a total of 223 consecutive patients underwent CRS/HIPEC for suspected CRPM. Of these, 25 patients (11.2%) had no histologically confirmed peritoneal metastasis in the abnormal tissue removed at CRS/HIPEC and were therefore excluded. A further three cases (1.3%) were excluded as their peritoneal tumour had originated from a separate non-colorectal malignancy (one patient exhibited a primary peritoneal tumour and two patients had peritoneal metastasis with an immunohistochemistry profile suggestive of origin from ovarian carcinoma). One hundred ninety-five patients with histologically confirmed CRPM at CRS/HIPEC were therefore included in this study, as outlined in Fig. 1. The median follow-up time for these patients was 34.7 months (range 5.4–184.9 months).

Genomic biomarker testing was available for the following genes: *KRAS* (*n* = 114), *NRAS* (*n* = 85), *BRAF* (*n* = 44), *PIK3CA* (*n* = 15) and *MMR* (*n* = 21). Testing of the *NRAS* gene was only ever done in combination with *KRAS*. Altogether, including results obtained from either primary or metastatic tumour, the rate of mutation was: 45.6% for *KRAS* (*n* = 52), 3.5% for *NRAS* (*n* = 3), 13.6% for *BRAF* (*n* = 6), 13.3% for *PIK3CA* (*n* = 2) and 14.3% for *MMR* (*n* = 3). Three patients with *KRAS* mutation had alteration present in another gene: one patient had concomitant *BRAF* mutation and two other patients possessed mutation in *PIK3CA*.

### Patient Demographics

Demographics for patients included within this study are presented in Table 1. The median age was 59.6 years (range 21.9–81.5 years) and 55.9% of patients were female. Primary tumour sites were distributed throughout the colon with caecum (29.2%) and sigmoid colon (27.2%) as the most common sites. Tumours originated in the rectum below the peritoneal reflection in 9.2% of patients. All but one patient had information available regarding their TNM staging, 74.4% of tumours were staged as pT4, 75.3% were node positive (N1 or N2), 26.2% of tumours were mucinous adenocarcinomas and 6.2% had signet ring cell features. One patient had a mixed adeno-neuroendocrine carcinoma. Peri-operative outcomes for patients treated with CRS/HIPEC are also presented in Table 1. The majority of patients received HIPEC with mitomycin C (68.2%), with the remainder receiving oxaliplatin (31.8%). The median PCI score overall was 9 (range 0–33), with a complete cytoreduction (CC0) achieved in 145 patients (74.4%). There were no significant differences in any of the baseline demographics and perioperative data between RAS mutant and RAS wild-type groups (*p* > 0.05; Table 1).

**Table 1:** Summary of patient demographics and peri-operative data for patients treated with CRS/HIPEC (*n* = 195) according to RAS status

Characteristic	RAS mut	RAS wt	RAS n/a
	(*n* = 55)	(*n* = 59)	(*n* = 81)
	*N* (%)	*N* (%)	*N* (%)
Age (years)			
Median	59.5	56.7	60.2
Range	31.7–75.6	21.9–75.9	29.5–81.5
Sex			
Male	26 (47.2)	25 (42.4)	35 (43.2)
Female	29 (52.7)	34 (57.6)	46 (56.8)
Primary site			
Unspecified area of colon	1 (1.8)	–	–
Caecum	14 (25.4)	14 (23.7)	29 (35.8)
Ascending colon	5 (9.1)	8 (13.6)	7 (8.6)
Hepatic flexure	1 (1.8)	2 (3.4)	3 (3.7)
Transverse colon	3 (5.5)	6 (10.2)	2 (2.5)
Splenic flexure	2 (3.6)	1 (1.7)	5 (6.2)
Descending colon	4 (7.3)	4 (6.8)	4 (4.9)
Sigmoid colon	18 (32.7)	20 (33.9)	15 (18.5)
Rectosigmoid	2 (3.6)	1 (1.7)	6 (7.4)
Rectum	5 (9.1)	3 (5.1)	10 (12.3)
T stage			
Tx	–	1 (1.7)	1 (1.2)
T2	1 (1.8)	2 (3.4)	3 (3.7)
T3	11 (20)	12 (20.3)	18 (22.2)
T4	43 (78.2)	44 (74.6)	58 (71.6)
*N* stage			
N0/Nx	15 (27.2)	8 (13.6)	24 (29.6)
N1	21 (38.2)	24 (40.7)	29 (35.8)
N2	19 (34.5)	27 (45.8)	27 (33.3)
Tumour composition			
Adenocarcinoma	33 (60)	43 (72.9)	55 (67.9)
Mucinous adenocarcinoma	19 (34.5)	12 (20.3)	20 (24.7)
Adenocarcinoma with signet ring cells +/− mucin	3 (5.5)	3 (5.1)	6 (7.4)
Mixed adenoneuroendocrine carcinoma	–	1 (1.7)	–
Differentiation			
Well	4 (7.3)	2 (3.4)	4 (4.9)
Moderate	39 (70.9)	45 (76.3)	51 (63)
Poor	10 (18.2)	9 (15.3)	19 (23.5)
N/a	2 (3.6)	3 (5.1)	7 (8.6)
Extramural vascular invasion			
Present	24 (43.6)	35 (59.3)	37 (45.7)
Not identified	25 (45.4)	17 (28.8)	28 (34.6)
N/a	6 (10.9)	7 (11.9)	16 (19.8)
HIPEC agent			
Oxaliplatin	18 (32.7)	15 (25.4)	29 (35.8)
Mitomycin C	37 (67.3)	44 (74.6)	52 (64.2)
PCI score			
Mean (SD)	11.5 (7.1)	11.4 (8.0)	10.1 (8.3)
Median (range)	11 (2–28)	9 (0–33)	7 (0–31)
PCI Category			
< 11	26 (47.3)	29 (49.2)	48 (59.3)
11–15	20 (36.4)	19 (32.2)	15 (18.5)
> 15	9 (16.4)	9 (15.3)	15 (18.5)
N/a	–	2 (3.4)	3 (3.7)
CC score			
CC0	41 (74.5)	42 (71.2)	62 (76.5)
CC1	8 (14.5)	8 (13.6)	7 (8.6)
CC2–3	6 (10.9)	9 (15.3)	12 (14.8)

TNM stage unavailable for one patient

*CC* completeness of cytoreduction, *Mut* mutant, *N-stage* nodal stage,* T-stage* tumour stage, *Wt* wild type, *N/a* not available

Pre- and post-operative SACT regimens for the patients included in this study are presented in Table 2. Chemotherapeutic treatment most commonly consisted of the following cytotoxic agents either alone or in combination: oxaliplatin, capecitabine, 5-fluorouracil (5FU) and irinotecan. One hundred fifty-seven patients received systemic chemotherapy before CRS/HIPEC with a median number of eight cycles completed (range 1–63). The 127 post-CRS/HIPEC patients received further systemic therapy with a median of 6.5 cycles (range 1–68). Anti-EGFR treatments (cetuximab, bevacizumab or Panitumumab) were used in 27 patients prior to CRS/HIPEC and 37 patients after CRS/HIPEC. A total of 16 patients underwent a repeat CRS/HIPEC procedure.

**Table 2:** Summary of systemic therapy pre- and post-CRS/HIPEC

No. of patients treated with systemic anti-cancer therapy	Pre-HIPEC		Post-HIPEC		
	Yes *n* = 157	No *n* = 38	Yes *n* = 127	No *n* = 59	N/A *n* = 9
*No. of cases/chemotherapy agent used*					
FOLFOX	88	–	37	–	–
FOLFIRI	23	–	70	–	–
CAPOX	36	–	23	–	–
CAPIRI	2	–	6	–	–
Mitomycin and capecitabine	0	–	6	–	–
Capecitabine only	26	–	24	–	–
Irinotecan only	0		14		
Oxaliplatin only	0		4		
5FU only	16	–	10	–	–
Mitomycin only	0	–	1	–	–
Long course chemoXRT	6	–	7	–	–
Bevacizumab	14	–	12	–	–
Cetuximab	10	–	21	–	–
Panitumumab	3	–	3	–	–
TAS-102	1	–	20	–	–
Aflibercept	0	–	3	–	–
Ralitrexed	0	–	2	–	–
Regorafenib	0	–	1	–	–
Other Trial Drug	0	–	8	–	–
Median no. of cycles received	8.0 (range 1–63)	–	6.6 (range 1–68)	–	–
Redo HIPEC	–	–	16	–	–

### Overall Survival Data

**Fig. 2:**
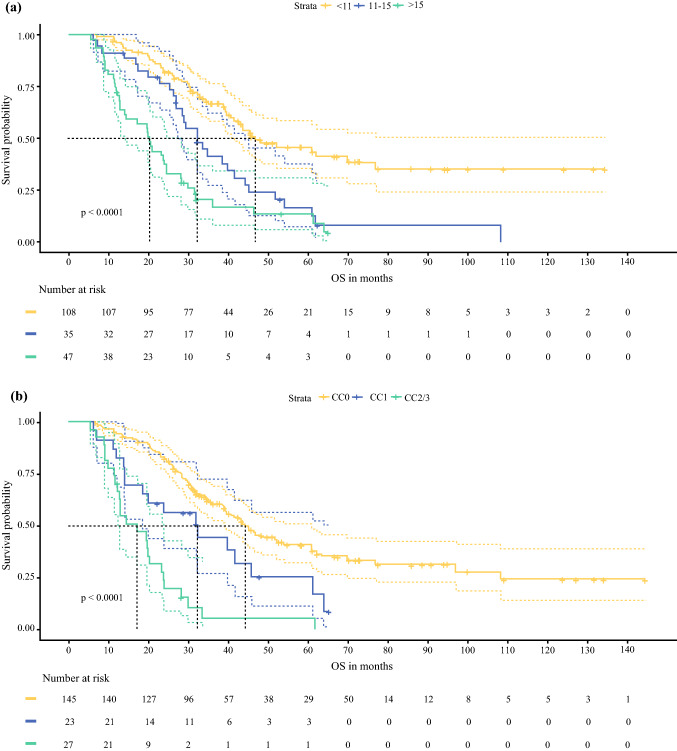
Kaplan–Meier plots showing overall survival in patients treated with CRS/HIPEC according based on **a** PCI and **b** CC scores

**Fig. 3.**
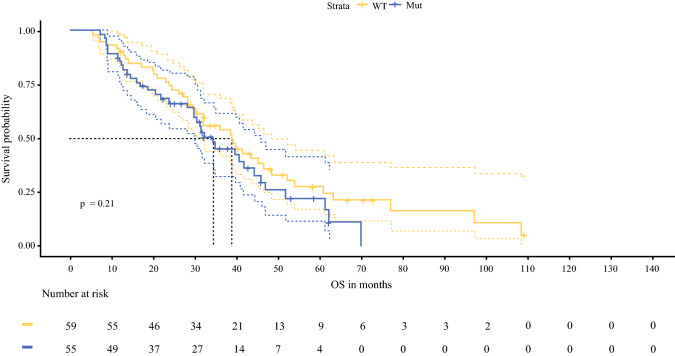
Kaplan–Meier plots showing overall survival in patients treated with CRS/HIPEC according based on *RAS* biomarker status

**Fig. 4.**
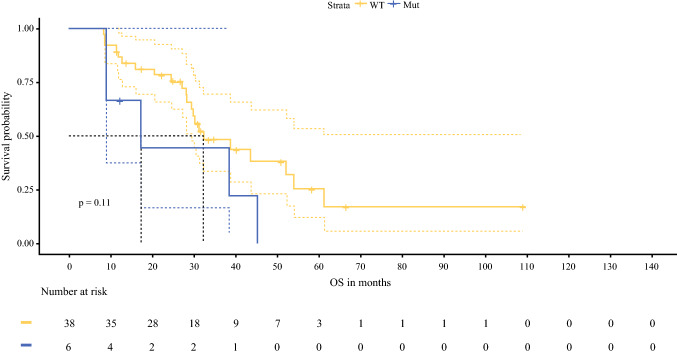
Kaplan–Meier plots showing overall survival in patients treated with CRS/HIPEC based on *BRAF* biomarker status

The median follow-up for all patients was 34.7 months (range 5.4–184.9 months), with median OS of 38.7 months (95% CI 32.4–44.9 months). The median OS was 46.7 months for patients with PCI < 11 (95% CI 31.0–62.5 months), 32.2 months for PCI 11–15 (95% CI 26.1–38.2 months) and 20.2 months for patient with PCI > 15 (95% CI 15.9–24.5 months), (*p* < 0.0001; Fig. 2). The median OS was 44.2 months for those with CC = 0 (95% CI 37.3–51.0 months), 32.2 months for CC = 1 (95% CI 16.8–47.5 months) and 17.0 months for CC = 2/3 (95% CI 8.7–25.3 months), *p* < 0.0001; Fig. 2). The median OS for *RAS* wild-type patients was 38.3 months (95% CI 30.4–46.3 months) compared with 34.7 months (95% CI 23.8–45.6 months) for *RAS* mutant tumours and was not statistically significant (*p* = 0.21; Fig. 3). Forty-four patients who underwent *BRAF* V600 testing showed a reduction in median OS from 32.1 months (95% CI 19.7–44.5 months) for *BRAF* wild-type tumours versus 17.2 months (95% CI 1.0–33.4) for *BRAF* mutant tumours (*p* = 0.109; Fig. 4).

### Univariate Survival Analysis

Positive nodal involvement with N1 status (HR: 1.703, 95% CI 1.024–2.831, *p* = 0.004), N2 status (HR: 2.561, 95% CI 1.559–4.206, *p* < 0.005), presence of extramural venous invasion (EMVI; HR: 1.718, 95% CI 1.146–2.576, *p* = 0.009), PCI score (HR: 1.082, 95% CI 1.058–1.106, *p* < 0.0005), CC score (HR: 5.898, 95% CI 3.647–9.537, *p* < 0.0005), SACT post-HIPEC (HR: 2.111, 95% CI 1.344–3.316, *p* = 0.001) and *NRAS* gene mutation (HR: 4.196, 95% CI 1.257–14.013, *p* = 0.020) were identified as significant negative predictors of OS. *KRAS*, *BRAF* and SACT prior to HIPEC; HIPEC chemotherapy type (oxaliplatin or mitomycin); poor and/or mucinous tumour differentiation; and the presence of signet cells in tumour did not significantly predict OS (*p* > 0.05).

### Multivariate Survival Analysis

The factors which demonstrated significance within univariate analysis were all included in the multivariate regression. Following a backwards stepwise approach, CC score and RAS status were consistent within the final multivariate Cox model, as shown in Table 3. CC-score > 1 (HR: 7.599, 95% CI 3.402–16.974, *p* < 0.0001) was a statistically significant negative predictor of OS, however, RAS mutation status (HR: 1.682, 95% CI 0.995–2.843, *p* = 0.052) did not significantly predict OS. Addition of the RAS/CC interaction to the multivariate Cox regression demonstrated no effect on the statistical model.

**Table 3:** Multivariate Cox proportional hazards model for overall survival (OS)

Variable	Hazard ratio	95% CI	*p* value
CC score			< 0.0001
CC0	Ref		
CC1	1.626	0.804–3.288	0.176
CC2–3	7.599	3.402–16.974	< 0.0001
RAS status			
Wild-type	Ref		
Mutant	1.682	0.995–2.843	0.052

## Discussion

Patients undergoing CRS/HIPEC for CRPM with curative intent are highly selected for the procedure based on tumour distribution and volume (lower radiologically derived PCI score). This is because complete clearance of the tumour (CC = 0) at surgery has been shown to be one of the most important determinants of outcome, and patients are selected based on the likelihood of this being achieved (74.4% in our study).^7^ Unsurprisingly, both high PCI and CC scores were found to significantly reduce OS. Another factor that impacts on the decision to offer CRS/HIPEC is a patient’s response to SACT (in this study 80.5% of patients received SACT at some point before CRS/HIPEC) and selection based on this introduces a potential bias towards patients with favourable tumour biology (responsive to chemotherapy or slow rate of progression). In this study, SACT after CRS/HIPEC was found to be a significant negative determinant of outcome, but SACT before CRS/HIPEC was not (univariate analysis). This may reflect the fact that mainly those with favourable response from SACT prior to CRS/HIPEC were likely to be selected. Post-CRS/HIPEC SACT is reserved for patients who are a poorer prognostic group (residual disease, high nodal burden, high risk of systemic metastatic disease).

In this study, 48.2% of the patients had right-sided primary tumours (originating from the caecum to the distal transverse colon), 42.1% had left-sided tumours (splenic flexure to sigmoid colon) and 9.2% had rectal cancers. Analysis of prognosis based on tumour location and biomarker status was not possible owing to the small sample size. Patients presented with pT4 tumours in 74.4% of cases and 75.4% had node-positive CRC, as would be expected in patients with advanced CRC presenting with peritoneal metastases. The biomarker mutation rates in this study (*KRAS* = 45.6%, *NRAS* = 3.5%, *BRAF* = 13.6%, *PIK3CA* = 13.3% and *MMR* = 14.3%) are also as expected from the literature.^15^ Biomarker mutation status was not a criterion for declining CRS/HIPEC in our institution, and our findings demonstrate that RAS mutation (*KRAS* and *NRAS* wild type) does not predict OS in our CRPM patients selected for CRS HIPEC with curative intent. Whilst the limited data available from several groups has suggested the use of this genomic marker to predict outcome from CRS/HIPEC and even proposed it be used as part of a scoring system to select patients for surgery,^9,11^ our findings support two larger recent studies that have not shown RAS mutation to predict outcome in this patient group.^12,13^ In our study, *BRAF* V600 mutation status showed a trend towards predicting poorer OS, however, the sample size was too small to draw definitive conclusions.

This study had several limitations which should be noted. First, despite being one of the largest series of CRPM patients undergoing CRS/HIPEC with characterised genomic biomarker status, the sample size was small. This is a limitation of the literature to date and will be addressed as genomic testing becomes more widespread. Second, during the study period there was the potential for heterogeneity in genomic testing with not all biomarkers being routinely tested. Extended panel and next-generation sequencing have only become routinely available in the UK National Health Service (NHS) in the last 5 years. Finally, this was a retrospective review of patients and therefore factors such as percentage tumour content in the samples, primary versus peritoneal metastasis sampling and DNA/RNA extraction could not be standardised.

This study reinforces the recent evidence that RAS mutation status should not be used to select patients for CRS/HIPEC or to predict outcome following the procedure. This is a patient group who in the majority receive SACT prior to CRS/HIPEC, which introduces a significant selection bias. A significant proportion also go on to receive post-CRS/HIPEC chemotherapy. Whether RAS mutated tumours, which are known to benefit less from SACT, should be selected for CRS/HIPEC before systemic treatment remains to be demonstrated. PCI and CC scores remain the key determinants used to select patients for CRS/HIPEC.
